# Pre-Injury Antiplatelet Therapy and Risk of Adverse Outcomes after Traumatic Brain Injury: A Systematic Review and Meta-Analysis

**DOI:** 10.1089/neur.2022.0042

**Published:** 2022-08-10

**Authors:** François Mathieu, Armaan K. Malhotra, Jerry C. Ku, Frederick A. Zeiler, Jefferson R. Wilson, Farhad Pirouzmand, Damon C. Scales

**Affiliations:** ^1^ Division of Neurosurgery, Department of Surgery, Management and Evaluation, Dalla Lana School of Public Health, University of Toronto, Toronto, Ontario, Canada.; ^2^ Interdepartmental Division of Critical Care Medicine, Management and Evaluation, Dalla Lana School of Public Health, University of Toronto, Toronto, Ontario, Canada.; ^3^ Department of Human Anatomy and Cell Science, University of Manitoba, Winnipeg, Manitoba, Canada.; ^4^ Section of Neurosurgery, Department of Surgery, Rady Faculty of Health Sciences, University of Manitoba, Winnipeg, Manitoba, Canada.; ^5^ Biomedical Engineering, Faculty of Engineering, University of Manitoba, Winnipeg, Manitoba, Canada.; ^6^ Centre on Aging, University of Manitoba, Winnipeg, Manitoba, Canada.; ^7^ Division of Anaesthesia, Department of Medicine, Addenbrooke's Hospital, University of Cambridge, Cambridge, United Kingdom.; ^8^ Department of Neurosurgery, St. Michael's Hospital, Toronto, Ontario, Canada.; ^9^ Division of Neurosurgery, Sunnybrook Health Sciences Centre, Toronto, Ontario, Canada.; ^10^ ICES, Toronto, Ontario, Canada.; ^11^ Department of Critical Care Medicine, Sunnybrook Health Sciences Centre, Toronto, Ontario, Canada.; ^12^ Institute of Health Policy, Management and Evaluation, Dalla Lana School of Public Health, University of Toronto, Toronto, Ontario, Canada.

**Keywords:** antiplatelet therapy, intracranial hemorrhage, mortality, traumatic brain injury

## Abstract

There is an increasing number of trauma patients presenting on pre-injury antiplatelet (AP) agents attributable to an aging population and expanding cardio- or cerebrovascular indications for antithrombotic therapy. The effects of different AP regimens on outcomes after traumatic brain injury (TBI) have yet to be elucidated, despite the implications on patient/family counseling and the potential need for better reversal strategies. The goal of this systematic review and meta-analysis was to assess the impact of different pre-injury AP regimens on outcomes after TBI. In accordance with Preferred Reporting Items for Systematic Reviews and Meta-Analyses (PRISMA) guidelines, the OVID Medline, Embase, BIOSIS, Scopus, and Cochrane databases were searched from inception to February 2022 using a combination of terms pertaining to TBI and use of AP agents. Baseline demographics and study characteristics as well as outcome data pertaining to intracerebral hematoma (ICH) progression, need for neurosurgical intervention, hospital length of stay, mortality, and functional outcome were extracted. Pooled odds ratios (ORs) and mean differences comparing groups were calculated using random-effects models. Thirteen observational studies, totaling 1244 patients receiving single AP therapy with acetylsalicylic acid or clopidogrel, 413 patients on dual AP therapy, and 3027 non-AP users were included. No randomized controlled trials were identified. There were significant associations between dual AP use and ICH progression (OR, 2.81; 95% confidence interval [CI], 1.19–6.61; *I*^2^, 85%; *p* = 0.02) and need for neurosurgical intervention post-TBI (OR, 1.61; 95% CI, 1.15–2.28; *I*^2^, 15%; *p* = 0.006) compared to non-users, but not between single AP therapy and non-users. There were no associations between AP use and hospital length of stay or mortality after trauma. Pre-injury dual AP use, but not single AP use, is associated with higher rates of ICH progression and neurosurgical intervention post-TBI. However, the overall quality of studies was low, and this association should be further investigated in larger studies.

## Introduction

Population aging has resulted in more patients having indications for antithrombotic therapy to prevent cardio- and cerebrovascular complications.^1–3^ At the same time, these shifting demographics have also been associated with a rising number of older patients presenting with blunt traumatic brain injury (TBI), predominantly attributable to falls.^4^ Pre-injury use of oral vitamin K antagonists has been associated with worse outcomes after TBI, but recent studies suggest that newer generations of direct oral anticoagulants may not be associated with an increased risk.^5–18^ The attributable risk conferred by antiplatelet (AP) therapy remains uncertain, with conflicting results from several studies.^5,10,14,19–36^

Moreover, many of the studies exploring the link between AP use and outcome after TBI have pooled different AP regimens together, making it difficult to delineate specific drug-outcome associations.^18,21,31,37–40^ Specifically, the attributable risk of pre-injury use of single-AP versus dual-antiplatelet therapy (DAPT) on outcomes after TBI is poorly understood. We therefore conducted a systematic review and meta-analysis to evaluate the relationship between pre-injury use of commonly prescribed AP regimens and clinical outcomes, including rates of intracranial hemorrhage (ICH) progression, need for surgical intervention, hospital of length of stay, mortality, and global functional outcome.

## Methods

### Data source and search strategy

We conducted a systematic review and meta-analysis adhering to the Preferred Reporting Items for Systematic reviews and Meta-Analyses (PRISMA) statement and Meta-analyses of Observational Studies in Epidemiology (MOOSE) reporting guidelines.^41,42^ Our study and search protocols were developed *a priori* and pre-registered with PROSPERO. We searched the OVID Medline, Embase, BIOSIS, Scopus, and Cochrane databases from inception to February 2022 using a combination of terms pertaining to TBI and use of AP agents. The full search strategy is detailed in the Supplementary Materials. Reference lists from the included publications and relevant review articles were screened manually to identify additional studies potentially meeting our eligibility criteria.

### Study selection and eligibility criteria

We used the Patients, Intervention, Comparison, and Outcomes (PICO) framework to develop our research question and eligibility criteria: Population = adult TBI patients, Intervention/Exposure = pre-injury use of AP therapy, Control group = patients not on any antithrombotic agents, Outcome = progression of ICH on repeat neuroimaging, need for neurosurgical intervention, total hospital length of stay, all-cause in-hospital mortality, mortality at 30 days, 6 months, or 1 year, death attributable to intracranial injury, and functional outcome at discharge, 6 monthss, and 1 year determined using the Glasgow Outcome Scale (GOS), Extended Glasgow Outcome Scale (GOSE), or modified Rankin Scale (mRS). We included retrospective studies, prospective cohort studies, case-control studies with sample sizes of at least 5 patients, and randomized controlled trials that included adult TBI patients and which evaluated the relationship between pre-injury use of AP therapy and pre-specified clinical outcomes.

Adult TBI patients were defined as persons ≥18 years of age admitted with a diagnosis of blunt TBI based on radiographical evidence of ICH, with or without concomitant extracranial injuries. Studies that pooled event rates for various AP or antithrombotic agents together and did not report treatment-stratified outcomes were excluded. Non-English articles, review articles, conference abstracts, and studies without a control group were also excluded. Articles identified with our search strategy were screened in a two-step process. A first filter based on article title and abstract was independently performed by two reviewers (F.M., J.C.K.) to identify articles potentially meeting our eligibility criteria. A second filter was independently conducted by the first and second authors (F.M., A.K.M.) based on the full article manuscripts. Discrepancies between the two authors were resolved by consensus or a third reviewer as necessary.

### Data extraction

Study characteristics, patient demographics, and outcome measures were extracted using a standardized electronic form and database. Specific data fields included study type, sample size, population, specific AP agent studied, exclusion criteria, percentage of female participants, mean GCS score, mean age, and pre-specified outcome measures. Dichotomous outcome measures were abstracted as event/non-event rates for each of the AP subgroups and for non-users. Continuous variables were recorded as subgroup means and standard deviations. Unfavorable outcome was defined as scores on the GOS of 1–3, GOSE 1–4, or mRS 3–6.^43–45^ Early mortality was defined as all-cause in-hospital or 30-day mortality rates, selected as the longest time point reported in the individual studies.

### Statistical analysis

We applied random-effect models using the Mantel-Haenszel method to calculate pooled odds ratios (ORs) for dichotomous variables and the inverse variance method to calculate mean differences (MDs) for continuous outcome data. We used the τ^2^, *I*^2^, and χ^2^ measures to assess for statistical heterogeneity between studies. We also performed sensitivity analyses for each of the outcomes of interest by sequentially excluding each study from the meta-analysis to test the robustness of pooled effect-size estimates. Data analyses were performed using Review Manager software (RevMan 5.4; The Cochrane Collaboration) and R (R Core Team [2016]; R: A language and environment for statistical computing; R Foundation for Statistical Computing, Vienna, Austria).

### Risk of bias assessment

Risk of bias for each of the included studies was assessed using the RTI item bank.^46^ Studies were categorized as being at low risk of bias, high risk of bias, or unclear risk of bias for each of the bias domains included in the RTI questionnaire. Funnel plots were constructed to assess risk of publication bias and small-study effects for each of the outcome of interest, and the rank-correlation and Egger's tests were performed for each comparison to detect significant asymmetry.

## Results

### Search results

**FIG. 1.**
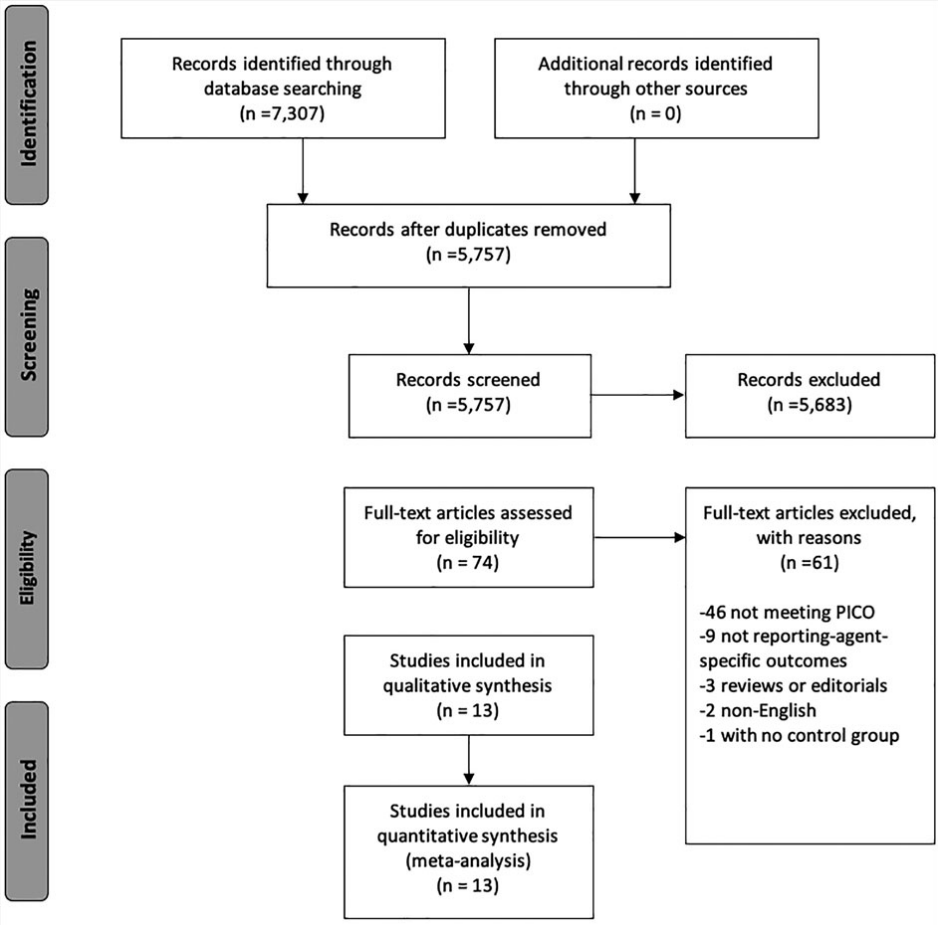
PRISMA flow diagram. PICO, Patients, Intervention, Comparison, and Outcomes; PRISMA, Preferred Reporting Items for Systematic Reviews and Meta-Analyses.

A PRISMA flow diagram providing an overview of our search results and screening process is provided as Figure 1. Our search strategy returned a total of 7307 articles. A total of 1550 duplicate titles were removed. Of the 5757 remaining articles included in the first filter, 5683 did not meet our eligibility criteria and were excluded. Seventy-four full-text articles were reviewed, and 61 were excluded for the following reasons: 46 studies did not meet our PICO criteria, nine studies did not report specific AP therapy results for any of the outcomes of interest, three were editorials or review articles, two articles were published in languages other than English, and one study did not include a control group. The remaining 13 articles were included in our systematic review and meta-analysis (Table 1).

**Table 1.** Study Characteristics of Included Studies

Author	Year	Study type	Sample size	Population	AP agents	Percent female	GCS	Age (mean ± SD)	Outcome measures	Conclusion
Fortuna	2008	RC	416	TBI ≥50 yr	ASA Clopidogrel DAPT	39.0%	12.0 ± 0.2	69.0 ± 1.0	Mortality Hospital LOS	Pre-injury use of clopidogrel, aspirin, or warfarin in older TBI patients is not associated with increased mortality.
Grandhi	2015	RC	1552	TBI ≥65 yr	ASA Clopidogrel DAPT	55.0%	15 [3–15]	79.9 ± 7.8	Mortality NSx intervention ICH progression Hospital LOS ICU LOS	Pre-injury use of warfarin, but not antiplatelet medications, influences survival and need for neurosurgical intervention in elderly TBI patients. ICH progression and morbidity are not affected.
Ivascu	2008	RC	109	TBI ≥50 yr	ASA Clopidogrel DAPT	44.0%	13.6 ± 2.8	77.0 ± 10.0	Mortality ICH progression	ASA or clopidogrel or both in elderly patients who have TBI resulting in ICH is associated with high mortality. However, the presenting GCS and initial grade of CT scan are most predictive of death.
Jones	2006	RC	1020 (46 with ICH)	TBI ≥50 yr	Clopidogrel	42.0%	NR	73	NSx intervention Rebleeding Mortality ICU LOS Hospital LOS	A higher percentage of patients in the clopidogrel group underwent cranial surgery, had episodes of rebleeds, and required transfusions of blood products than in the control group. Mortality and length of stay were comparable in both groups.
Joseph	2014	PPMC	144	TBI ≥18 yr	ASA	40.3%	15 [IQR, 14–15]	72.8 ± 11.7	Mortality Discharge GCS ICH progression NSx intervention	Low-dose aspirin therapy is not associated with progression of initial insult on repeat head CT or clinical deterioration.
Joseph	2014	PPMC	142	TBI ≥18 yr	Clopidogrel	34.0%	14 [3–15]	70.5 ± 15.1	Mortality Discharge GCS ICH progression NSx intervention	Pre-injury clopidogrel therapy is associated with progression of initial insult on repeat head CT and need for neurosurgical intervention.
Koiso	2021	RC	393	TBI ≥18 yr	ASA Clopidogrel other AP DAPT	32.8%	NR	75.0 [IQR, 64.0–81.0)	ICH progression 30-day mortality Neurological death mRS at discharge	Pre-injury antiplatelet use was not associated with more unfavorable outcome.
Mathieu	2020	PPMC	316	TBI ≥18 yr	ASA Clopidogrel other AP DAPT	35.0%	14 [3–15]	67.9 ± 12.2	ICH progression NSx intervention ICU LOS Hospital LOS Mortality Neurological death GOSE at 6 months	Pre-injury use of antithrombotic agents was associated with greater expansion of extra-axial lesions, higher rates of significant hemorrhagic progression, and higher risk of delayed ICH, but this was not associated with worse clinical course or functional outcome.
Mina	2002	RCC	74	TBI ≥18 yr	ASA	41.0%	12.2 ± 3.4	74.5 ± 9.6	Hospital LOS Mortality	Pre-injury ASA use was associated with a 4- to 5-fold higher risk of death after intracranial injury compared to controls.
Probst	2020	PC	9070 (532 with ICH)	TBI ≥18 yr	ASA Clopidogrel DAPT	39.0%	NR	54.8 [IQR, 34.7–74.3]	Risk of ICH NSx intervention	Patients receiving pre-injury warfarin or a combination of aspirin and clopidogrel were at increased risk for significant intracranial injury, but not those receiving aspirin alone.
Scotti	2020	RC	1365 (564 with ICH)	TBI ≥65 yr	ASA Clopidogrel DAPT	50.8%	14.1 ± 2.3	79.5 ± 8.1	Presence of ICH NSx intervention GOSE at discharge Mortality	Elderly trauma patients on ATs, especially combination therapy, are at elevated risk of ICH and poor outcomes compared with those not on AT therapy. Use of single-AP therapy was not associated with mortality; however, the combination of aspirin and clopidogrel was.
Sumiyoshi	2017	RC	934	TBI ≥60 yr	Single AP (any) DAPT	35.8%	12.0 ± 3.6	73.3 ± 8.6	Mortality ICH progression Hospital LOS GOS at discharge	The outcome of patients with TBI, who were on AP agents, may be determined by the severity of pre-existing conditions.
Wong	2008	RCC	289	TBI ≥18 yr	Clopidogrel	36.3%	14.7	66.7	Mortality LOS Discharge disposition	TBI patients on clopidogrel may have increased long-term disability and fatal consequences when compared with patients who are not on these drugs or on other antithrombotics.

AC, anticoagulant; AP, antiplatelet; ASA, acetylsalicylic acid; AT, antithrombotic; CT, computed tomography; DAPT, dual antiplatelet therapy; GCS, Glasgow Coma Scale; GOSE, Extended Glasgow Outcome Scale; ICH, intracranial hemorrhage; ICU, intensive care unit; LOS, length of stay; mRS, modified Rankin Scale; NR, not reported; NSx, neurosurgery; PC, prospective cohort; PPMC, prospective propensity-matched cohort; RC, retrospective cohort; RCC, retrospective case-control; SD, standard deviation; TBI, traumatic brain injury.

### Study characteristics

The included studies were published between 2002 and 2021. Seven studies were retrospective cohort studies, two were retrospective case-control studies, three were propensity-matched cohort studies, and one was a prospective observational cohort study. No randomized controlled trials were identified. Approximately half of the studies included all adult TBI patients ≥18 years of age, and the remainder focused on older TBI patients ≥50 to ≥65 years. Across all studies, mean subject age ranged from 67 to 80 years. All studies reported on AP regimens consisting of either acetylsalicylic acid (ASA), clopidogrel, or ASA combined with clopidogrel and included a non-user group as controls. The AP subgroup outcome data were not reported in the published manuscript for one study, but the raw data were accessible to the authors and therefore included in the meta-analysis.^28^ Most studies did not report on timing of AP agent discontinuation or resumption post-TBI or on adverse events that might be related to withholding of AP therapy.

### Qualitative synthesis of included studies

No studies reported differences in rates of ICH progression or need for neurosurgical interventions comparing the ASA and control groups. Pre-injury use of clopidogrel was associated with greater ICH progression compared to the control group in only one of three studies that reported on hematoma expansion for this subgroup,^26^ and one of six studies showed a higher requirement for neurosurgical intervention among clopidogrel users compared to the control group. No studies reported a significant association between clopidogrel therapy and increased length of hospital stay.^35^ Dual AP use, however, was associated with more ICH progression compared to controls in three of four studies and higher rates of neurosurgical interventions in one of five studies.^28,33,34^

Most (8 of 11) studies that reported mortality rates did not observe significant associations with pre-injury use of any AP agent regimen.^10,22–28^ One small study reported a 4- to 5-fold risk of death for subjects on ASA compared to non-users.^29^ Another study reported increased mortality and long-term disability rates for patients taking clopidogrel before their TBI.^35^ Only two of the seven studies that reported on mortality rates for the subgroup of patients on DAPT detected a significant association.^10,22,23,27,28,33,34^ Rates of platelet transfusions after TBI in the different patient subgroups varied significantly across studies and are summarized in the Supplementary Materials.

### Meta-analysis of study outcomes

#### Intracerebral hematoma progression

**FIG. 2.**
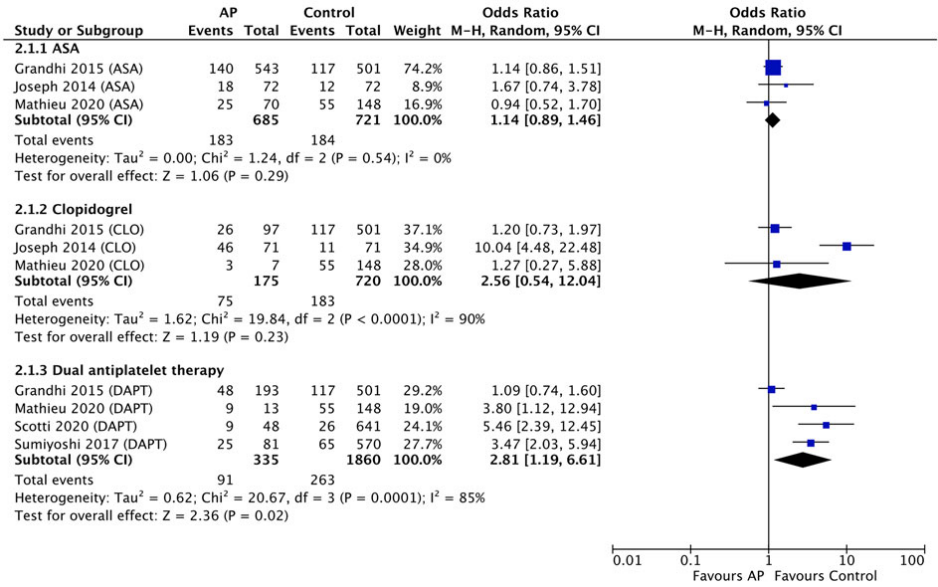
Forest plot of meta-analysis comparing ICH progression in antiplatelet users versus non-users. AP, antiplatelet; ASA, acetylsalicylic acid; CI, confidence interval; CLO, clopidogrel; DAPT, dual antiplatelet therapy; ICH, intracerebral hematoma.

Six studies reported on rates on ICH progression for the various AP regimens, totaling 1195 AP users and 2003 non-users. Pooling individual study data revealed a significant association between use of DAPT and ICH progression (OR, 2.81; 95% confidence interval [CI], 1.19–6.61; *I*^2^, 85%; *p* = 0.02; Fig. 2). In contrast, single-agent therapy with either ASA or clopidogrel was not associated with a significantly increased risk of hemorrhagic progression (OR, 1.14; 95% CI, 0.89–1.46; *I*^2^, 0%; *p* = 0.29 and OR, 2.56; 95% CI, 0.54–12.04; *I*^2^, 90%; *p* = 0.23).

### Neurosurgical intervention

**FIG. 3.**
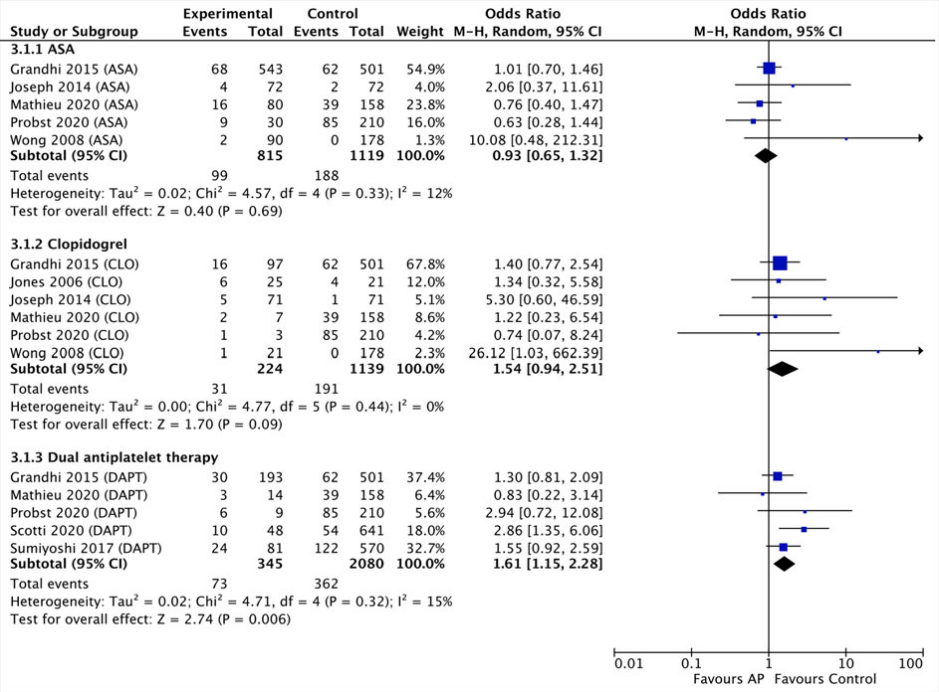
Forest plot of meta-analysis comparing rates of neurosurgical intervention in antiplatelet users versus non-users. ASA, acetylsalicylic acid; CI, confidence interval; CLO, clopidogrel; DAPT, dual antiplatelet therapy.

Nine studies reported rates of neurosurgical intervention in a total of 1384 AP users and 2422 non-users. Dual-agent, but not single-agent, AP therapies was more likely to require a neurosurgical procedure in comparisons to non-AP users (dual AP OR, 1.61; 95% CI, 1.15–2.28; *I*^2^, 15%, *p* = 0.006; ASA OR, 0.93; 95% CI, 0.65–1.32; *I*^2^, 12%, *p* = 0.69; clopidogrel OR, 1.54; 95% CI, 0.94–2.15; *I*^2^, 0%, *p* = 0.09; Fig. 3).

### Length of stay

Pre-injury ASA use was associated with slightly shorter hospital length of stay (LOS) compared to controls after pooling of study data (MD, −0.30; 95% CI −0.50 to −0.11; *I*^2^, 2%; *p* = 0.002). There was no significant association between clopidogrel or dual-AP use and total hospital LOS compared to controls (clopidogrel MD, 0.18; 95% CI, −0.35 to 0.71; *I*^2^, 7%, *p* = 0.50; DAPT MD, −2.35; 95% CI, −5.25, 0.54; *I*^2^, 90%, *p* = 0.11). There were insufficient studies reporting on intensive care unit (ICU) LOS for pooled statistical analysis.

### Mortality

**FIG. 4.**
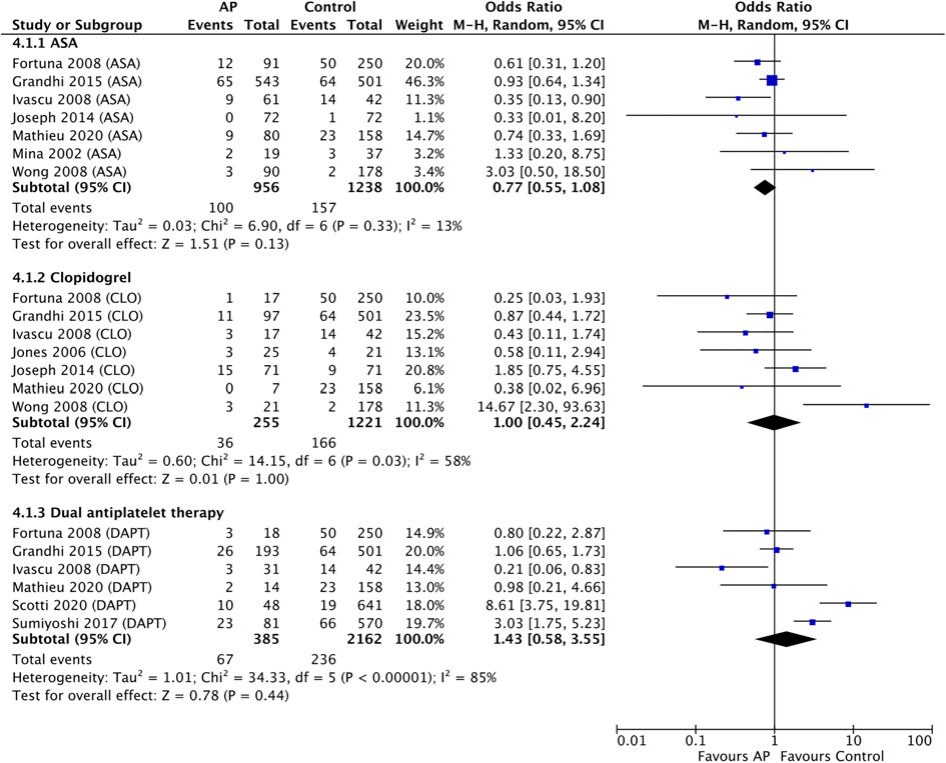
Forest plot of meta-analysis comparing mortality in antiplatelet users versus non-users. AP, antiplatelet; ASA, acetylsalicylic acid; CI, confidence interval; CLO, clopidogrel; DAPT, dual antiplatelet therapy.

Mortality data were available in 11 studies comparing a total of 1596 AP users with 2541 non-users. Pooled early mortality rates were not statistically different between ASA, clopidogrel, and dual-AP users compared to non-users (ASA OR, 0.77; 95% CI, 0.55–1.08; *I*^2^, 13%, *p* = 0.13; clopidogrel OR, 1.00; 95% CI, 0.45–2.24; *I*^2^, 58%, *p* = 1.00; DAPT OR, 1.43; 95% CI, 0.58–3.55; *I*^2^, 85%, *p* = 0.44; Fig. 4). There were insufficient studies reporting on mortality rates at 6 and 12 months after injury for pooled statistical analysis.

### Functional outcome

**FIG. 5.**
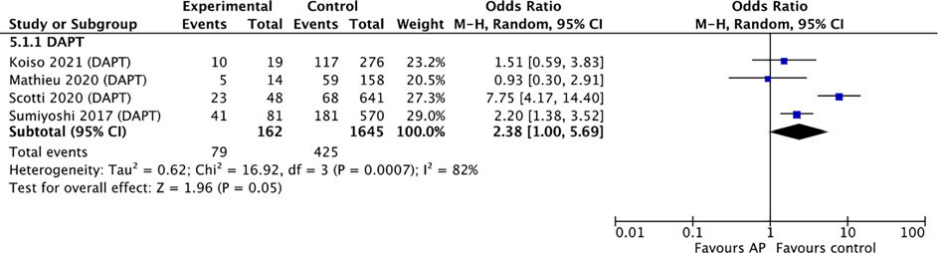
Forest plot of meta-analysis comparing functional outcomes in dual antiplatelet therapy users versus non-users. CI, confidence interval; DAPT, dual antiplatelet therapy.

Only four of the included studies provided data on global functional outcome using standardized measures, and some studies combined results for different AP therapy regimens because of small subgroup sizes.^27,28,33,34^ As a result, we only were able to analyze functional outcome for dual-AP users compared to controls, which did not have a significant association and wide confidence intervals (OR, 2.38; 95% CI, 1.00–5.69; *I*^2^, 82%; *p* = 0.05; Fig. 5).

### Risk of bias

**FIG. 6.**
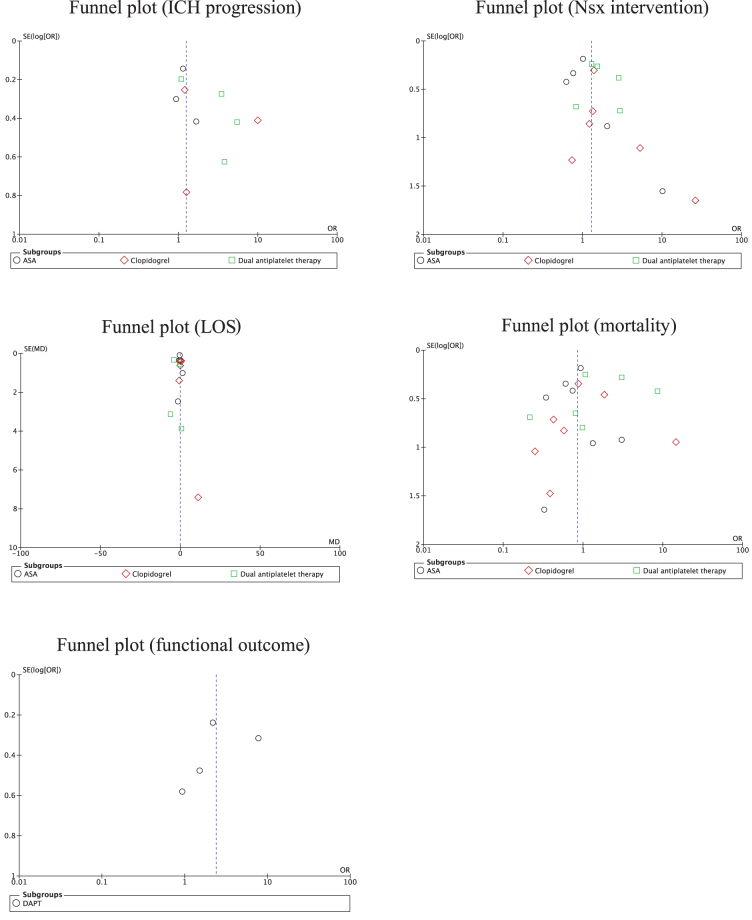
Funnel plots exploring risk of publication bias for outcome of interest. ASA, acetylsalicylic acid; DAPT, dual antiplatelet therapy; ICH, intracerebral hematoma; LOS, length of stay; Nsx, neurosurgery.

Most studies were deemed at moderate to high risk of bias owing to their small sample sizes, observational nature, and lack of standardized measures for some of the outcomes of interest and inconsistent lengths of follow-up across participants. A summary of the RTI data bank bias assessment for each study is available in the Supplementary Materials. When considering risk of bias across studies, there was significant variation in the degree of heterogeneity observed with *I*^2^ values ranging from 0% to 90%. The funnel plots to assess for publication bias and small study effects are provided as Figure 6. The rank-correlation and Egger's regression tests did not reveal any significant asymmetry (Kendall's τ coefficient, −0.33 to 0.40 and Z = −1.19 to 1.31, respectively; all *p* > 0.05) despite some asymmetry on visual inspection.

## Discussion

To our knowledge, this is the largest systematic review and meta-analysis to evaluate the differential effects of specific pre-injury AP regimens on outcomes after TBI. Our pooled analyses showed that DAPT comprising a combination of ASA plus clopidogrel was associated with an increased risk of ICH progression and need for neurosurgical intervention, but not with increased early mortality or rate of unfavorable outcome. Conversely, single-AP therapy with ASA pre-injury did not appear to confer a higher risk of ICH progression, need for neurosurgical intervention, or other adverse clinical outcomes. Patients receiving clopidogrel monotherapy also did not demonstrate a statistically significant increase in risk of adverse outcomes, although wider confidence intervals prevent us from drawing robust conclusions for this subgroup.

A previous smaller meta-analysis evaluated the relationship between aspirin or clopidogrel use and mortality after TBI and did not find any significant association.^19^ However, as the authors highlighted, this study was limited by a small number of included studies (*n* = 5) and small overall sample size to detect clinically important differences. A limitation of studies in this area is that many have combined patients taking different AP agents together, or even combined AP and anticoagulant users into a single group to maximize statistical power.^31,39,40^ Other studies found that crude associations between pre-injury use of AP therapies and increased mortality after TBI were no longer apparent after adjusting for age, GCS score, pre-injury disability, and radiological severity.^18^

There is strong pharmaco- and physiological rationale to believe that different agents (or multi-agent combinations) cause a varying degree of alteration in hemostatic pathways and respond differently to reversal attempts.^47,48^ A better understanding of the relationship between commonly prescribed regimens and risk of complications would not only be of prognostic value, but could also potentially provide an impetus for the development of more targeted reversal strategies and for consideration of alternative antithrombotic strategies in patients at high risk of falls or other trauma mechanisms. Our analysis suggests that dual-AP users are at particularly high risk of complications and worse outcomes after TBI. This is consistent with previous research indicating that the incremental reduction in thromboembolic events achieved with DAPT comes with a significantly increased risk of bleeding.^49^

### Limitations

Our systematic review and meta-analysis has several important limitations. First, given the low number of articles meeting our inclusion criteria and the moderate sample sizes of the included studies, our study is likely underpowered to conclusively rule out associations between some of the AP subgroups and the outcomes of interest. Most of the included studies were retrospective and did not perform multi-variate adjustment to account for other relationships that could impact clinical outcome measures—for example, severity of TBI and pre-injury disability. Most included studies reported all-cause mortality rather than attributable death from TBI and did not consider the potential for competing risk between rates of ICH progression and death. In addition, though we described the reversal strategies used in each study where available, there were insufficient data available for any pooled analyses of this information. The majority of studies also did not differentiate between low- and full-dose ASA, although there is evidence that both dosage regimens may exert a similar degree of AP effect.^50–52^

We focused our review on the most commonly prescribed AP therapies and are unable to comment on some of the less frequently prescribed AP agents. We also were unable to directly compare the attributable risks of specific AP therapies, given that all included studies only compared AP therapy users to non-users. Finally, our review summarizes the incremental risk of ICH progression and mortality after TBI that is associated with AP therapies, but does not consider the potential benefits of prescribing these agents across entire populations of eligible patients.

## Conclusion

Our systematic review and meta-analysis suggest that pre-injury DAPT, but not single-agent AP therapy, is associated with an increased risk of hemorrhagic progression and need for neurosurgical intervention after TBI. These findings should be confirmed and further quantified in larger future studies, but provide a starting point for shared decision making when counseling elderly patients about the risks and benefits of these treatments.

## Supplementary Material

Supplemental data

## Abbreviations Used

AP: antiplatelet
ASA: acetylsalicylic acid
CI: confidence interval
DAPT: dual antiplatelet therapy
GOS: Glasgow Outcome Scale
GOSE: Extended Glasgow Outcome Scale
ICH: intracerebral hematoma
ICU: intensive care unit
LOS: length of stay
MD: mean difference
mRS: modified Rankin Scale
OR: odds ratio
PICO: Patients, Intervention, Comparison, and Outcomes
TBI: traumatic brain injury
