# Supplementary material for: Pre-Injury Antiplatelet Therapy and Risk of Adverse Outcomes after Traumatic Brain Injury: A Systematic Review and Meta-Analysis
Source: Neurotrauma Rep. 2022 Aug 10;3(1):308–20. doi: 10.1089/neur.2022.0042 (PMC9438446; doi:10.1089/neur.2022.0042)
Supplement: Supplemental data [file Suppl_Data.zip › SupplementalData1.docx]

**Supplemental digital content: search terms**

1. traumatic brain injury.mp.
2. traumatic brain injury.tw.
3. TBI.mp.
4. TBI.tw.
5. head injury.mp.
6. head injury.tw.
7. head trauma.mp.
8. head trauma.tw.
9. brain trauma.mp.
10. brain trauma.tw.
11. brain injury.mp.
12. brain injury.tw.
13. cerebral trauma.mp.
14. cerebral trauma.tw.
15. cerebral injury.mp.
16. cerebral injury.mp.
17. intracranial hemorrhage.mp.
18. intracranial hemorrhage.tw.
19. intracranial bleeding.mp.
20. intracranial bleeding.tw.
21. cerebral contusion.mp.
22. cerebral contusion.tw.
23. anticoagulant.mp.
24. anticoagulant.tw.
25. antiplatelet.mp.
26. antiplatelet.tw.
27. antithrombotic.mp.
28. antithrombotic.tw.
29. adenosine diphosphate receptor inhibitor.mp.
30. adenosine diphosphate receptor inhibitor
31. glycoprotein inhibitor.mp.
32. glycoprotein inhibitor.tw.
33. GP IIb/IIIa inhibitor.mp.
34. GP IIb/IIIa inhibitor.tw.
35. aspirin.mp.
36. aspirin.tw.
37. acetylsalicylic acid.mp.
38. acetylsalicylic acid.tw.
39. clopidogrel.mp.
40. clopidogrel.tw.
41. ticlodipine.mp.
42. ticlodipine.tw.
43. prasugrel.mp.
44. prasugrel.tw.
45. ticagrelor.mp.
46. ticagrelor.tw.
47. cilostazol.mp.
48. cilostazol.tw.
49. dipyridamole.mp.
50. dipyridamole.tw.
51. abciximab.mp.
52. abciximab.tw.
53. tirofiban.mp.
54. tirofiban.tw.
55. eptifibatide.mp.
56. eptifibatide.tw.
57. dual antiplatelet therapy.mp.
58. dual antiplatelet therapy.tw.
59. 1 or 2 or 3 or 4 or 5 or 6 or 7 or 8 or 9 or 10 or 11 or 12 or 13 or 14 or 15 or 16 or 17 or 18 or 19 or 20 or 21 or 22
60. 23 or 24 or 25 or 26 or 26 or 27 or 28 or 29 or 30 or 31 or 32 or 33 or 34 or 35 or 36 or 37 or 38 or 39 or 40 or 41 or 42 or 43 or 44 or 45 or 46 or 47 or 48 or 49 or 50 or 51 or 52 or 53 or 54 or 55 or 56 or 57 or 58
61. 59 and 60

**Primary Data Sources:** OVID Medline, Embase, BIOSIS, Scopus and Cochrane Databases
