# Supplementary material for: Pre-Injury Antiplatelet Therapy and Risk of Adverse Outcomes after Traumatic Brain Injury: A Systematic Review and Meta-Analysis
Source: Neurotrauma Rep. 2022 Aug 10;3(1):308–20. doi: 10.1089/neur.2022.0042 (PMC9438446; doi:10.1089/neur.2022.0042)
Supplement: Supplemental data [file Suppl_Data.zip › SupplementalData2.docx]

**Reversal strategies used in the included studies**

| **Study** | **Reversal information** |
| --- | --- |
| Fortuna et al. | Not reported |
| Grandhi et al. | Platelet transfusion: 68.3% ASA group, 83.5% clopidogrel group, 85.5% DAPT group |
| Ivascu et al. | Platelet transfusion: 36.6% of subjects received a platelet transfusion |
| Jones et al. | Platelet transfusion: 12% of clopidogrel group, none in the control group |
| Joseph et al. | Platelet transfusion: 21% of ASA group, 4.2% of control group |
| Joseph et al. | Platelet transfusion: 71% of clopidogrel and 51% of control |
| Koiso et al. | None of the patients on AP therapy received a platelet transfusion |
| Mathieu et al. | Platelet transfusion: 14.3% of ASA group 28.6% of clopidogrel group |
| Mina et al. | Not reported |
| Probst et al. | Not reported |
| Scotti et al. | Platelet transfusion 4.2% of DAPT group Platelet transfusion + desmopressin: 10.4% of DAPT group |
| Sumiyoshi et al. | Platelet transfusion 4.9% of DAPT group, 2.1% of non-AP group |
| Wong et al. | Platelet transfusion: 21.1% of ASA group, none in clopidogrel group |

*AP, antiplatelet; DAPT, dual antiplatelet therapy*
