# Supplementary material for: Pre-Injury Antiplatelet Therapy and Risk of Adverse Outcomes after Traumatic Brain Injury: A Systematic Review and Meta-Analysis
Source: Neurotrauma Rep. 2022 Aug 10;3(1):308–20. doi: 10.1089/neur.2022.0042 (PMC9438446; doi:10.1089/neur.2022.0042)
Supplement: Supplemental data [file Suppl_Data.zip › SupplementalData3.docx]

**Supplemental digital content: summary of risk of bias assessments**

| **Reference** | **Inclusion/Exclusion Criteria** | **Recruiting** | **Power** | **Performance Bias** | **Blinding** | **Valid Measures of Outcome** | **Follow Up Length** | **Attrition** | **Harms Reporting** | **Analytic methods** | **Confounders Considered** | **Overall Bias Assessment** |
| --- | --- | --- | --- | --- | --- | --- | --- | --- | --- | --- | --- | --- |
| Fortuna 2008 | H | L | L | U | H | L | H | L | L | H | H | H |
| Grandhi 2015 | L | L | L | U | H | L | H | L | L | L | L | L |
| Ivascu 2008 | L | L | L | U | H | L | H | L | L | H | L | L |
| Jones 2006 | H | L | L | U | H | L | H | L | L | H | H | H |
| Joseph 2014 | L | L | L | U | L | L | H | L | L | L | L | L |
| Joseph 2014* | L | L | L | U | L | L | H | L | L | L | L | L |
| Koiso 2021 | L | L | L | U | H | L | L | H | L | L | L | L |
| Mathieu 2020 | L | L | L | U | L | L | L | L | L | L | L | L |
| Mina 2002 | L | L | H | U | H | H | H | H | L | H | H | H |
| Probst 2020 | L | L | L | U | H | L | H | L | L | L | L | L |
| Scotti 2020 | L | L | L | U | H | L | H | H | L | L | L | L |
| Sumiyoshi 2017 | L | L | L | U | H | L | L | H | L | L | L | L |
| Wong 2008 | L | L | H | U | H | L | L | L | L | L | H | L |
| *clopidogrel study |  |  |  |  |  |  |  |  |  |  |  |  |
